# Can high-flow nasal cannula reduce the risk of bronchopulmonary dysplasia compared with CPAP in preterm infants? A systematic review and meta-analysis

**DOI:** 10.1186/s12887-021-02881-z

**Published:** 2021-09-16

**Authors:** Sabrina de Jesus Brito, Sabrina Pinheiro Tsopanoglou, Endi Lanza Galvão, Franciele Angelo de Deus, Vanessa Pereira de Lima

**Affiliations:** 1 Physiotherapy Department, Postgraduate Program in Rehabilitation and functional performance, Universidade Federal dos Vales do Jequitinhonha e Mucuri, Campus JK - Diamantina/MG, Rodovia MGT 367 - Km 583, n° 5.000 Alto da Jacuba, Diamantina, Minas Gerais 39100-000 Brazil; 2 Physiotherapy Departmentat, Universidade Federal dos Vales do Jequitinhonha e Mucuri, Diamantina, Minas Gerais Brazil; 3 Graduate Program in Dentistry, Faculty of Biological and Health Sciences, Universidade Federal dos Vales do Jequitinhonha e Mucuri, Diamantina, Minas Gerais Brazil

**Keywords:** Nasal cannula, Nasal continuous positive airway pressure, Infant premature, Premature birth

## Abstract

**Background:**

Bronchopulmonary dysplasia (BPD) is a chronic lung disease that affects the premature lung, and to reduce its incidence has been used non-invasive ventilatory support, such as continuous positive airway (CPAP) and high-flow nasal cannula (HFNC). Thus, the objective of this review was to assess whether the use of high flow nasal cannula (HFNC) compared to continuous positive airway pressure (CPAP) decreases the risk of bronchopulmonary dysplasia (BPD) in premature newborns.

**Methods:**

The protocol was registered (Prospero: CRD42019136631) and the search was conducted in the MEDLINE, PEDro, Cochrane Library, CINAHL, Embase, and LILACS databases, and in the clinical trials registries, until July 2020. We included randomized clinical trials comparing HFNC versus CPAP use in premature infants born at less than 37 weeks of gestational age. The main outcome measures were the development of BPD, air leak syndrome, and nasal injury. The methodological quality of the included studies was assessed using the Cochrane risk of bias tool and the GRADE system was used to summarize the evidence recommendations. Meta-analyses were performed using software R.

**Results:**

No difference was found between HFNC or CPAP for the risk of BPD (RR: 1.10; 95% CI: 0.90–1.34), air leak syndrome (RR: 1.06; 95% CI: 0.52–2.14), and nasal trauma (RR: 2.00; 95% CI: 0.64–6.25), with a very low level of evidence.

**Conclusion(s):**

The HFNC showed similar results when compared to CPAP in relation to the risk of BPD, air leak syndrome, and nasal injury. In the literature, no randomized clinical trial has been found with BPD as the primary outcome to support possible outcomes.

**Supplementary Information:**

The online version contains supplementary material available at 10.1186/s12887-021-02881-z.

## Background

The bronchopulmonary dysplasia (BPD) is a chronic lung disease that results from an imbalance between lung injury, inflammation, repair and healing in the developing lung [1, 2]. Until 2018, mid-2019, the definition and classification made for BPD proposed by the National Institute of Child Health and Human Development (NICHD) and the Network Vermont-Oxford it was based in the premature infant’s need to receive supplemental oxygen at 36 weeks of post-menstrual age (PMA) or for more than 28 days of life [1, 3].

Considering the importance of predicting clinical respiratory outcomes and the neurodevelopment of BPD throughout childhood, several updates and changes in the definition of the disease are being proposed. Thus, according to the current definition proposed, BPD is the result of the combination of immaturity of lung development, injury, inflammation, repair and healing. Premature infants at greatest risk of developing BPD are those under 32 weeks of gestational age, with parenchymal lung disease with radiological image and need for supplemental oxygen for at least 3 days, to maintain peripheral oxygen saturation between 90 and 95%, at 36 weeks post-menstrual gestational age (PMA) [2, 4].

To reduce the incidence of BPD, a significant increase in the use of non-invasive ventilatory support, such as continuous positive airway pressure (CPAP), which provides stability to the newborn’s airways, increasing the gas exchange area and decreasing respiratory work, thus reducing the need for invasive mechanical ventilation have been used [5–7]. New modalities of non-invasive ventilation strategies include the use of the high-flow nasal cannula (HFNC) [6, 8], whose benefits include decreased airway resistance and better gas exchange, reducing respiratory overload [9–12].

A previous systematic review [13] compared the use of CPAP and HFNC in relation to the safety and efficacy of devices, as forms of primary intervention shortly after birth, or after extubation, including studies published until December 2018. In the study, the BPD outcome was secondary and despite the presented study having performed meta-analyzes, the review did not assess the quality of the evidence presented and new studies were published since December 2018.

In the same sense, a more recent systematic review was published in order to compare four different noninvasive respiratory support treatments (bilevel positive airway pressure (BiPAP), noninvasive positive pressure ventilation (NIPPV), HFNC and CPAP) in a network meta-analysis. However, BPD was evaluated as a combined outcome with mortality [14].

## Methods

This systematic review was to assess whether the use of HFNC compared to CPAP, decreases the risk of BPD in premature newborns, and was carried out according to PRISMA statement [15] (see Additional file 1) and the recommendations of the Cochrane Handbook [16]. The protocol was previously registered in the International Prospective Registry of Systematic Reviews (Prospero: CRD 42019136631).

The clinical question was formulated using the PICO strategy (P: patient, problem or population, I: intervention, C: comparison and O: outcome), as follows: **P:** premature newborns (born with gestational age less than 37 weeks); **I:** HFNC; **C:** CPAP; **O:** risk of BPD, as defined by National Institute of Child Health and Human Development (NICHD) and the Vermont-Oxford Network [1–3]. Secondary outcomes were the development of air leak syndrome and nasal injury.

Inclusion criteria comprised randomized controlled trials (RCT) that have as intervention the HFNC compared with the use of CPAP in premature newborns defined as born with gestational age less than 37 weeks, and studies reporting development of BPD as an outcome. Regarding the primary BPD effect, it was considered, for this review, such as the use of supplemental oxygen for ≥28 days of life or 36 weeks’ postmenstrual age (PMA). Nasal injury and air leak syndrome (pneumothorax, pneumomediastinum and pulmonary interstitial emphysema) were searched as possible side effects related to the interventions.

Studies were excluded if the sample size was less than or equal to 10 participants, if the patients were already diagnosed with BPD, or if they were protocols, abstracts, editorials, comments, presentations in congress and animal studies.

An electronic search of the published literature was conducted until July 2020, in the following databases: PubMed (MEDLINE), PEDro (Physiotherapy Evidence Database), Embase, Cochrane Library, CINAHL, and LILACS. The ClinicalTrials.gov was assessed to identify potential ongoing studies. No language restrictions were applied. The terms used in the search were the following keywords, according to the MeSH (Medical Subject Heading) terms: Bronchopulmonary Dysplasia, high flow nasal cannula, non-invasive ventilation, continuous positive airway pressure, nasal intermittent positive pressure ventilation, preterm infant and all similar terms. The Boolean operators used were “AND” and “OR” (see Additional file 2).

After searching the databases, the titles and abstracts of the articles were read by two authors independently (SJB and SPT) and all references identified by searches were exported to Mendeley Reference Manager Version 2.39.0, and duplicates were removed. The studies that could potentially meet the inclusion criteria for the review were identified at this stage and accessed in full. In case of disagreement, a third author (VPL) was consulted to obtain a consensus. All included studies were evaluated for qualitative and quantitative analysis, according to the data availability.

Participant and methodological characteristics were extracted from the included studies. Specifically, the following data were extracted: year of publication, country, therapy method, randomization, inclusion and exclusion criteria, BPD definition, and characteristic of the population enrolled in studies (gestational age, gender, birth weight, surfactant and associated diseases). Two reviewers independently carried out the data extraction (SJB and SPT).

The risk of bias in RCTs was evaluated by the Cochrane risk of bias tool [16], using the following six domains: random sequence generation (generation of the randomization sequence), allocation concealment, blinding of outcome assessment, incomplete outcome data, selective repotting, and other sources of bias such as the inclusion of preterm infants with specific gestational age, not including all preterm infants below 37 weeks of gestational age, and the absence of definition criteria for primary and secondary outcomes. The domain “blinding of patients and personnel” was not considered once it is not applicable to this type of study. All articles could have the following domain classifications: high risk of bias, low risk of bias, uncertain risk (without information for judgment).

The GRADE (Grading of Recommendations Assessment, Development and Evaluation) criteria was used to summarize the evidence recommendations [16]. In the present review, the evidence began with a high certainty. It was reduced to a level of inaccuracy when the sample analyzed was smaller than 200 participants; one level for medium risk and two levels for high risk assessed by the Cochrane bias tool [16]; and for a level of inconsistency if I2 ≥ 50% or asymmetric CI; one level when the outcomes were not similar and two levels when the metrics were not presented or diverged; at a level when publication bias is identified. Two independent reviewers (SPT and ELG) evaluated the quality of the evidence, and discrepancies were resolved by a third reviewer (VPL).

### Statistical analyses

The meta-analyses were performed using the R software version 3.6.2, with packages “meta” and “metafor” (R Foundation for Statistical Computing). Effect sizes were expressed as relative risk (RR) for dichotomous data and their 95% confidence intervals were calculated for analysis. The heterogeneity between the studies was tested by the inconsistency test *(I*^*2*^*)*: when the value of *I*^*2*^ was zero it indicates non-heterogeneity and the fixed effects model was used; a random-effects model was used in the presence of heterogeneity, considered when *I*^*2*^ was greater than 0%. Forest plots were created to present the results.

For the meta-analysis that included 10 or more studies, the *Begg’s* test was performed and visual analysis of the funnel plots were performed for testing the publication biases.

## Results

**Fig. 1:**
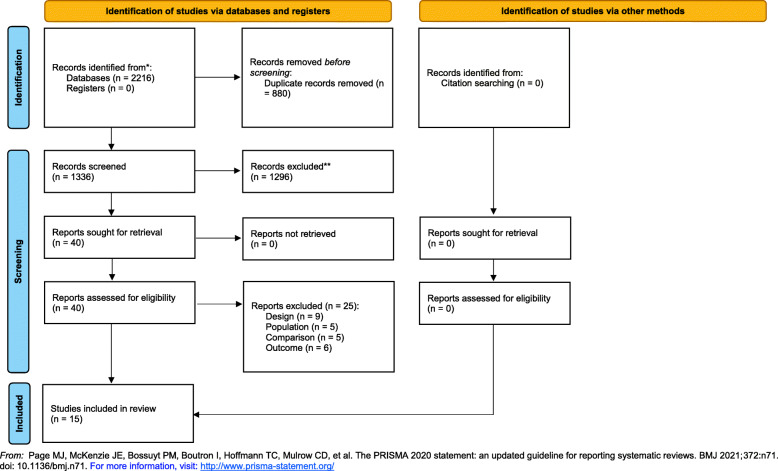
PRISMA flow diagram demonstrating included and excluded studies and reasons for exclusion

The electronic and hand searches identified a total of 2216 articles. After removal of duplicates, 1336 articles were screened of which 15 articles met the inclusion criteria [10, 11, 17–29]. The steps for selecting articles and the reason for exclusions are described in the PRISMA flow diagram (Fig. 1).

A total of 2038 neonates were analyzed in this study: 1014 preterm infants received treatment by HFNC and 1024 by CPAP [10, 11, 17–29].

In relation to the use of the intervention (HFNC) and its comparator (CPAP), most studies evaluated extubation failure in the first 72 h as the primary outcome [10, 11, 19, 21, 24, 26]. The others evaluated chronic lung disease after extubation [28], higher oxygen use requirement [29], treatment respiratory distress syndrome (RDS) primary [22, 24, 28], effectiveness and safety of HNFC and oral feeding [18, 20, 21, 27]. The main methodological characteristics of the included studies are shown in Additional file 3.

The gestational age of the study population varied from 26 to 33 weeks. Diseases such as early sepsis, patent ductus arteriosus, pneumothorax, intraventricular hemorrhage, infections necrotizing enterocolitis, and retinopathy of prematurity, were the most prevalent. The main characteristics of the participants are presented in Additional file 4.

Most studies defined BPD as using supplemental oxygen for 36 postmenstrual weeks [19, 22, 25, 28] while the others used the definition proposed by NICHD [10, 20, 27, 29] or presented no definition [7, 22, 24, 26, 28].

### Quality and heterogeneity

Most studies showed a high risk of bias in the blinding of outcome assessment domain. The domain that presented the highest risk of bias was in relation to the blinding of outcome assessment, and the domain with the least bias laugh was for incomplete outcome data (see Additional file 5).

**Fig. 2:**
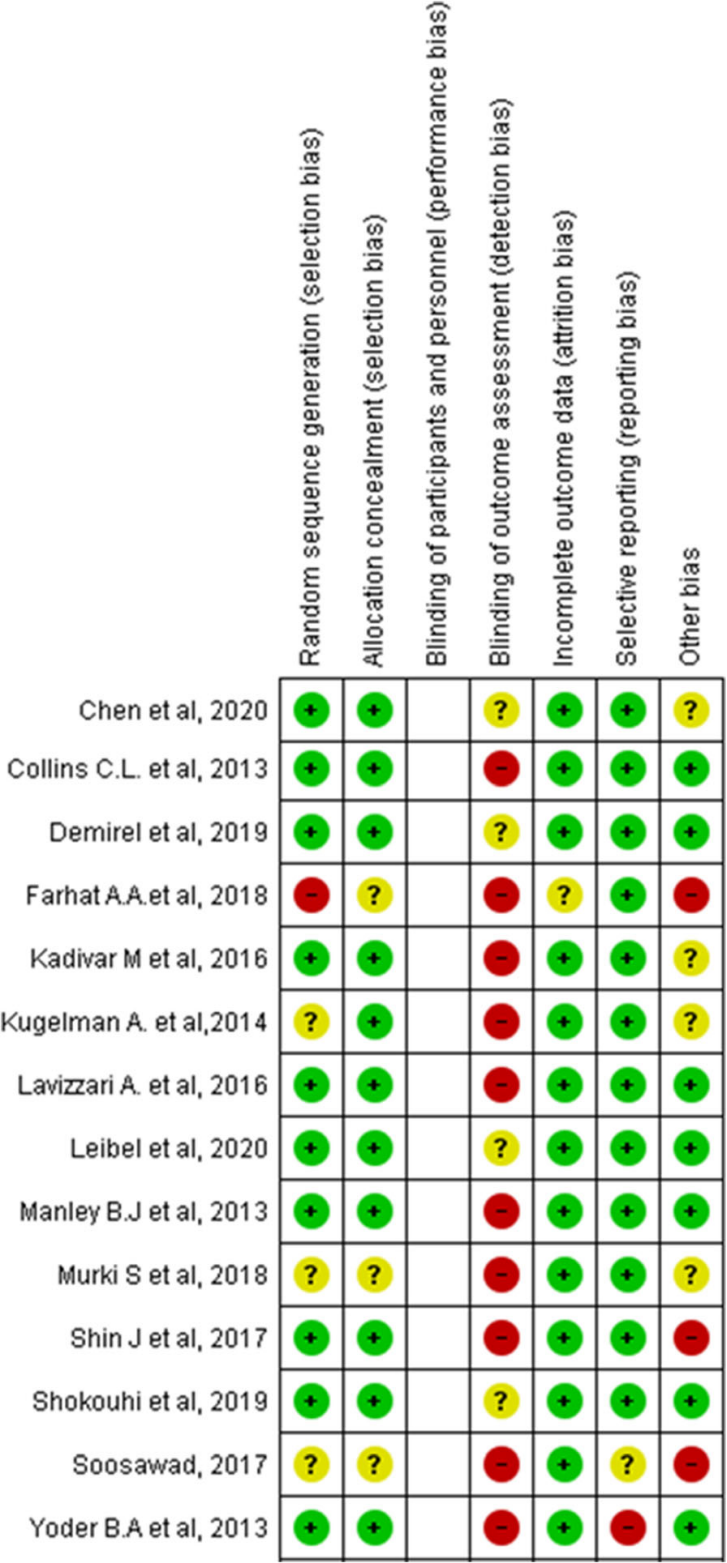
Assessment of the methodological quality and risk of bias of included clinical trial studies

The evaluation of the methodological quality and risk of bias of included clinical trial studies are shown in Fig. 2.

### Development of bronchopulmonary dysplasia

**Fig. 3:**
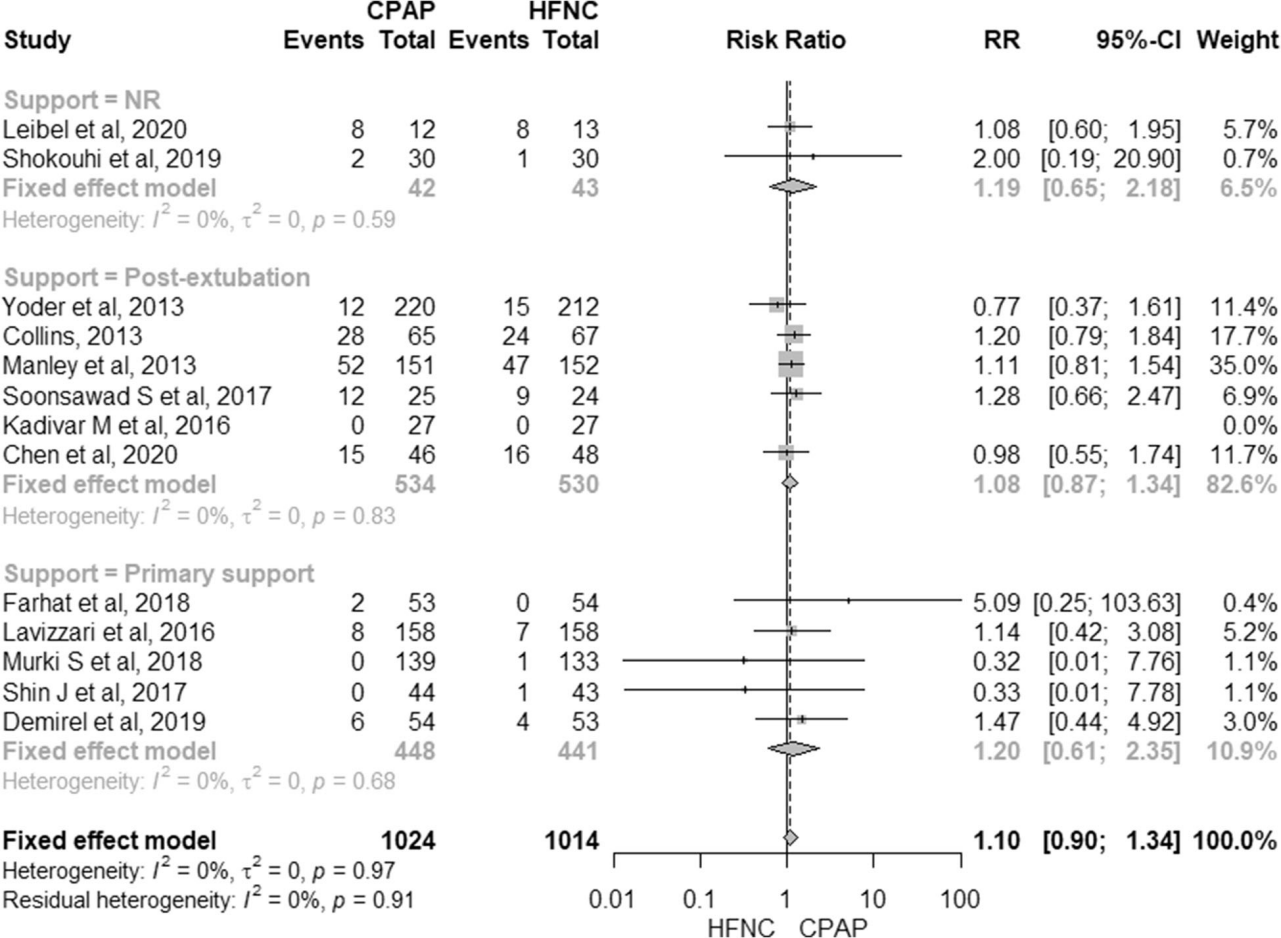
Meta-analysis for the risk of BPD

Thirteen studies comprising 2038 patients were included in the meta-analysis for BPD outcome [10, 11, 17–21, 24, 26–29]. There was no difference between the use of HFNC and CPAP in the development of BPD (RR: 1.10, 95% CI: 0.90–1.34, *I*^*2*^: 0%) and very low quality of evidence (see Additional file 6-GRADE). In the subgroup analysis according to the type of support (use of HFNC or CPAP in the post-extubation or as primary support, there is still no difference between the use of HFNC and CPAP in the development of BPD (Fig. 3).

### Development of air leak syndrome

**Fig. 4:**
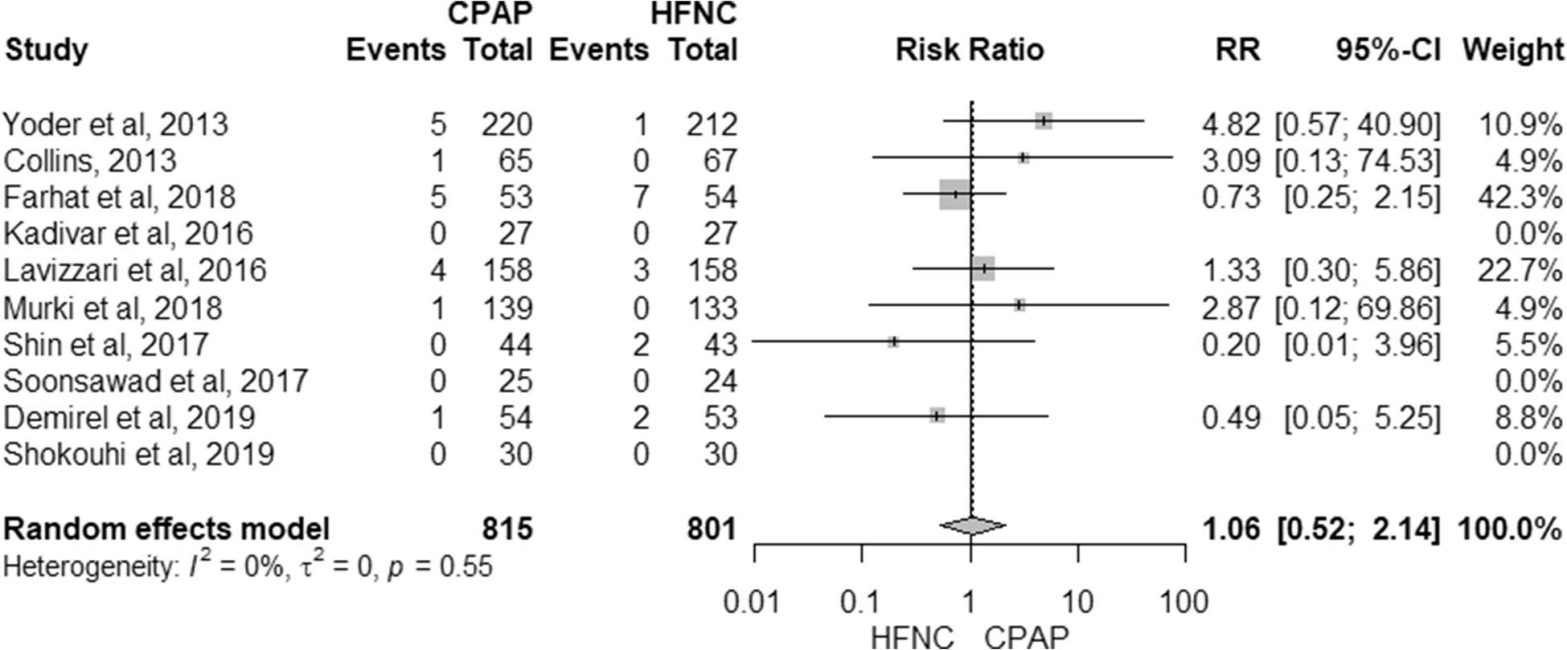
Meta-analysis for the risk of air leak syndrome

Ten studies presented data to be included in the meta-analysis related to air leak syndrome (15 in the HFNC group and 17 neonates in the CPAP group) [19–23, 28, 29]. There was no difference between the use of HFNC and CPAP and the development of the air leak syndrome (RR: 1.06, 95% CI: 0.52–2.14, *I*^*2*^: 0%) (Fig. 4), with very low quality of evidence (see Additional file 6-GRADE).

### Development of nasal injury

**Fig. 5:**
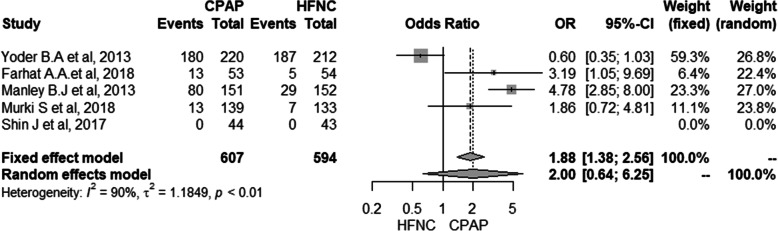
Meta-analysis for the risk of nasal injury

Five studies presented data to be included in the meta-analysis related to the absence of nasal injury (231 in the HFNC group and 305 neonates in the CPAP group) [10, 11, 19, 21, 27, 29]. In the same way, there was no difference between the use of HFNC and CPAP and the development of the nasal injury (RR: 2.00, 95% CI: 0.64–6.25, *I*^*2*^: 90%) (Fig. 5) with very low quality of evidence (see Additional file 6-GRADE).

**Fig. 6:**
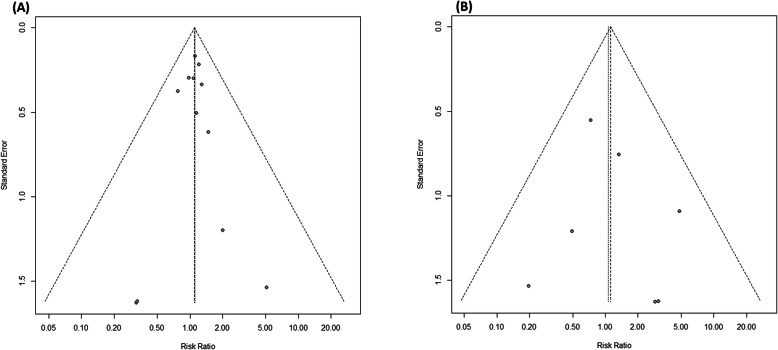
Funnel plot for the meta-analysis of relative risk for **a** BPD and **b** air leak syndrome outcomes

The visual inspection of the BPD (*p* = 0.6808) and air leak syndrome outcomes funnel plots did not show any substantial asymmetry (Fig. 6). It was not possible to perform the *Begg* test, as well as the funnel plot, as they did not present enough studies in the nasal injury meta-analyses.

## Discussion

Overall, the results of the present review demonstrate that there was no difference between the use of HFNC and CPAP in the risk of BPD. In addition, no significant difference was found between the use of HFNC and CPAP related to the development of the air leak syndrome (pneumothorax) and nasal injury.

Most studies eligible for the current systematic review had as main objective to assess the incidence of extubation failure with the use of HFNC [10, 11, 19, 21, 24–26], while other studies have evaluated the effectiveness of using HFNC as the primary treatment for the acute respiratory distress syndrome [22, 24, 28] and 4 studies had the primary objective of assessing the effectiveness and safety of HFNC [18, 20, 21, 27]. Only one study assessed the relationship between the use of HFNC and the incidence of BDP [28] and another one describes the use of HFNC with the greatest need for oxygen therapy [29].

BPD was first described 50 years ago and still lacks effective treatment and a comprehensive definition. Differently from the original description, BPD is currently characterized by chronic respiratory failure mainly in extreme neonates, together with changes in non-invasive respiratory support and the severity of long-term lung damage, which makes it more difficult to define the current condition. Limitations related to definitions include the inability to classify neonates who die before 36 weeks and possibly the use of HFNC with room air (21%) or very low flow with 100% oxygen makes some neonates not included in these definitions [2, 7]. However, keeping the criteria for defining the disease in just one specification can exclude relevant studies, restricting the results.

Even with the significant increase in the use of these non-invasive ventilation devices in the past two decades, there is evidence that incidence rates of BPD [30] remained unchanged. Studies suggest that these results may occur due to the excessive use of interventions associated with other risk factors for BPD, such as infections in the peri and postnatal period, contributing to premature lung injuries [30, 31].

CPAP is recommended worldwide, by the World Health Organization, as first-line therapy for the treatment of premature newborns with respiratory disorders since birth, significantly improving oxygenation, when compared to HFNC, which may justify its greater popularity [19, 32, 33]. The HFNC application systems, currently available, do not measure pressure in the airways, which can lead to the release of excessive pressure, contributing to the appearance of lung lesions and consequently contributing to the development of BPD [33]. Therefore, the clinical use of lower flow rates and adequate control of it leads to a reduction in this risk [34]. The results of the present study are in accordance with the present one, since there was no greater risk for the development of BPD with the use of HFNC.

The literature points out that nasal injury is a common complication in premature infants using CPAP, with a prevalence of 20 to 60% [34, 35]. Alternatives to prevent nasal trauma when using CPAP include the use of appropriately sized interfaces and dressings as protection for the skin [29, 35, 36]. Evidence indicates that the lower prevalence of injury to the nasal septum with the use of HFNC is due to the humidified and heated flow offered by the device, which reduces the inflammation of the upper airway epithelial cells [34, 37] and the injury to the nasal mucosa, in addition to fact that the HFNC interface is lighter and easier to install compared to CPAP [36–38].

The high heterogeneity presented in the results for the nasal lesion outcome can be justified by the fact that most of the studies included in the meta-analysis did not present a standardized metric for assessing the outcome, as described in the GRADE table (see Additional file 6), as well as different forms administration of ventilatory support and the lack of explanation of the sizes and types of interfaces used.

There is a concern about the use of HFNC and the risk of air leak syndrome due to high flows, as the pressure within the circuit cannot be measured, allowing the supply of high flows to the lower airways [18–38]. A recent systematic review published in 2019 demonstrated a reduction in the prevalence of air leak syndrome with HFNC use compared to CPAP, in premature neonates, as a post-extubation conduct [39]. Our results observed no difference in relation to the air leak syndrome, specifically the pneumothorax, in accordance with the results of a recent published network meta-analysis comparing both interventions [14].

The quality of the included studies determines the quality of the systematic review, which is why we conducted a review using strict quality assessment criteria in randomized clinical trial studies [15]. There was a high risk of bias and risk of uncertain bias in most studies. Another consideration in this sense is that is a lack of blindness in the participants and personnel due to the nature of the application of the intervention and the evaluator, factors that increase the chance of bias related to the included studies. Another limiting factor in the present study was the fact that we included preterm infants regardless of classification. It is known that one of the risk factors for BPD is extreme prematurity or very premature, that is, premature infants born with less than 32 weeks of gestational age. However, we chose to include all preterm infants since in the literature, RCTs that sampled only extremely preterm infants, or very preterm infants are scarce. The studies showed great variability in the protocols for the application of CPAP and HFNC, and there was also great variability in the BPD definitions. Regarding the criteria for the diagnosis of air leak syndrome and nasal injury, many studies did not present the metrics for diagnosis of the presented outcomes, which led to a higher level of observed indirect evidence, a factor that contributed to decrease the quality of the evidence. Thus, the evidence based on the included RCTs was of very low quality.

According our results, the effective prevention of BPD still remains a challenge, since the results found cannot be generalized for clinical application. Therefore, the choice between non-invasive ventilation devices, HFNC or CPAP, remains a matter of clinical judgment by the team, which must analyze what outcomes it intends to use with the device of choice.

## Conclusion

In this systematic review with meta-analysis, we highlight that the HFNC showed similar results when compared to CPAP in relation to the risk of BPD, air leak syndrome and nasal injury. In the literature, no RCT has been found with BPD as the primary outcome to support possible outcomes. It is recommended that further research should be undertaken in this field with higher methodological quality to support the expected results.

## Supplementary Information

**Additional file 1.** PRISMA 2009 Checklist.

**Additional file 2.** Search strategies.

**Additional file 3.** Main methodological characteristics of included studies.

**Additional file 4.** Characteristics of the participants enrolled in studies.

**Additional file 5.** Assessment of risk of bias of included clinical trial studies.

**Additional file 6.** GRADE scale.

## Data Availability

The data pertaining to the current analysis may be sent to the corresponding author, Sabrina Pinheiro Tsopanoglou (sabrina.pinheiro@ufvjm.edu.br).

## Abbreviations

BPD: Bronchopulmonary dysplasia
CPAP: Continuous positive airway pressure
HFNC: Flow nasal cannula
PMA: Post-menstrual gestational age
RCT: Randomized controlled trials
