# Supplementary material for: Can high-flow nasal cannula reduce the risk of bronchopulmonary dysplasia compared with CPAP in preterm infants? A systematic review and meta-analysis
Source: BMC Pediatr. 2021 Sep 16;21:407. doi: 10.1186/s12887-021-02881-z (PMC8444598; doi:10.1186/s12887-021-02881-z)
Supplement: Supplementary file 2 — Additional file 2. Search strategies. [file 12887_2021_2881_MOESM2_ESM.docx]

Additional file 2- Search strategies

| **Eletronic database** | **Set of terms (key terms)** |
| --- | --- |
| Pubmed | (non-invasive ventilation OR noninvasive **ventilation**OR Ventilation* OR non-invasive ventilations OR continuous positive airway pressure OR cpap OR biphasic continuous positive airway pressure OR bilevel continuous positive airway pressure OR nasal continuous positive airway pressure OR ncpap ventilation OR nasal intermittent positive pressure ventilation))) AND (flow high nasal cannula OR cannula, nasal OR nasal cannula OR cannulae, nasal OR cannulae)) AND (preterm infant OR infants OR premature OR premature infant OR preterm infants OR infant, preterm OR infants, preterm OR neonatal prematurity OR prematurity, neonatal) |
| BVS/LILACS | (premature$) AND (Cannula or Cánula or Cânula or Cânula Nasal) AND (Noninvasive Ventilation OR Ventilación no Invasiva OR Ventilação não Invasiva OR CPAP OR BILEVEL)  (infant or premature) AND (noninvasive ventilation) AND (cannula$)  (tw:(infant or premature)) AND (tw:(noninvasive ventilation)) AND (tw:( cannula)) |
| PEDro | Cannula*Noninvasive Ventilation*premature* |
| CIHNAL – EBSCO | (non-invasive ventilation OR noninvasive ventilation OR Ventilation* OR non-invasive ventilations OR continuous positive airway pressure OR cpap OR biphasic continuous positive airway pressure OR bilevel continuous positive airway pressure OR nasal continuous positive airway pressure OR ncpap ventilation OR nasal intermittent positive pressure ventilation) AND (flow high nasal cannula OR cannula, nasal OR nasal cannula OR cannulae, nasal OR cannulae) AND (preterm infant OR infants OR premature OR premature infant OR preterm infants OR infant, preterm OR infants, preterm OR neonatal prematurity OR prematurity, neonatal) |
| EMBASE | (('infant, premature'/exp OR 'infant, premature' OR (infant, AND ('premature'/exp OR premature))) AND 'high flow nasal cannula oxygen' AND 'noninvasive ventilation' OR (continuous AND positive AND pressure AND ventilation) OR (nasal AND intermittent AND positive AND pressure AND ventilation)) AND ([controlled clinical trial]/lim OR [randomized controlled trial]/lim) AND [2019-2020]/py |
