# Supplementary material for: Can high-flow nasal cannula reduce the risk of bronchopulmonary dysplasia compared with CPAP in preterm infants? A systematic review and meta-analysis
Source: BMC Pediatr. 2021 Sep 16;21:407. doi: 10.1186/s12887-021-02881-z (PMC8444598; doi:10.1186/s12887-021-02881-z)
Supplement: Supplementary file 3 — Additional file 3. Main methodological characteristics of included studies. [file 12887_2021_2881_MOESM3_ESM.docx]

**Additional file 3- Main methodological characteristics of included studies**

| **Year, Author** | **Country** | **Therapy method**  **(patients)** | **Randomized (Y/N)** | **Inclusion criteria** | **Exclusion criteria** | **BPD**  **definition** | **Type of support** |
| --- | --- | --- | --- | --- | --- | --- | --- |
| 2020, Chen J et al^11^ | China | HFNC: FiO2: 30%-40%, flow:4-6 /min, at 37°C.  CPAP: flow 4-8 L/min, PEEP 5-7 cmH2O, FiO2:40%, | Y | GA <32 and weight:<1,000 g; with SDR; invasive ventilation and within seven days after birth; prepared for tracheal extubation and a change to non-invasive ventilation. | Congenital airway mal formations, congenital diaphragmatic hernia, congenital lung dysplasia, tracheo esophageal fistula; life-threatening congenital mal formations. | NR | Post-extubation |
| 2020, Leibel S et al^17^ | Canadá | HFNC: 5-8 L/min.  CPAP:5-10 cm/H_2_O | Y | GA ≤28; IGCo=34/7; tolerating full enteral feeds and were not orally feeding | GA >28 weeks, extubated less than a week prior to randomization, on biphasic CPAP or NIPPV, or had significant congenital or neurological abnormalities. | NICHD | NR |
| 2019, Demirel G et al^18^ | Turquia | HFNC: 6L/min (max.8). FiO2 was adjusted to maintain SpO2 between 90 and 95%. CPAP: 6 cm/H_2_O (max. 7) | Y | GA ≤32 with an AIG and had spontaneous respiration. | Intubation after birth, congenital anomalies, intrauterine growth restriction, and perinatal asphyxia. | NR | Primary support |
| 2019, Shokouhi M et al^19^ | Iran | HFNC: 2-8 L/min  Oxygen flow (L/min) = 0.92 + (0.68 + weight [kg]); FiO2: 0.21-1.0  CPAP: 4 cm /H_2_O; FiO2:0.4. | Y | GA: 28 - 36 RDS affliction, NICU admission length of < 24 h, oxygen requirement with the FiO2 of more than 40 mmHg, and 5-min Apgar  Score: > 5. | Congenital heart defects or other  major congenital anomalies. | NR | NR |
| 2018, Murki S et al^20^ | Índia | HFNC: 1-7 L/min.  CPAP: pressures to SpO_2_:90%-95%. | Y | PT≥28 weeks GA; birth weight ≥1,000 g; SDR first 6h of birth | Infants with major mal formations and those intubated in the DR. | 36 weeks PMA | Primary support |
| 2018, Farhat A A et al^21^ | Iran | CPAP: 6 - 8cm/H_2_O  HFNC: 2 - 5 L/min | N | PT:28-34 weeks and 800-2500 g; | Severe asphyxia, major anomalies, < 800g or > 2500 g, GA <28 or> 34 weeks, congenital pneumonia, primary blood culture, parental dissatisfaction. | Supplemental oxygen more than 28 days | Primary support |
| 2017, Soonsawad S et al^22^ | Thailand | HFNC: 4 L/min  CPAP: pressure was set similar to the pressure of PEEP of ventilator | **Y** | Intubated, (GA) <32 weeks  and <1500 g | < 700 g, major congenital heart diseases, airway anomalies, lung hypoplasia, and neuromuscular disorders. | NR | Post-extubation |
| 2017, Shin J et al^10^ | Korea | HFNC: 3-7 L/min  CPAP: 4-7 cm/H_2_O | **Y** | Not meet the invasive respiratory support | GA <30 weeks or< 1,250 g; congenital anomalies of the upper airway tract, major congenital or chromosomal abnormalities, air leak or cardiovascular instability. | NICHD | Primary support |
| 2016, Lavizzari A et al^23^ | Italy | HNFC: 4 to 6 L/min  CPAP: 4 to 6 cm/H_2_O | Y | GA: 29- 36 weeks 6 days; mild to moderate SDR, and parental consent obtained | Severe RD; major congenital anomalies respiratory; or severe IVH. | NR | Primary support |
| 2016, Kadivar M et al^24^ | Irã | HFNC: 4 L/min  CPAP:5-8 cmH_2_O | Y | SDR, surfactant replacement therapy, extubated within one hour after INSURE method | Asphyxia, respiratory disease, major congenital anomalies, and had not needed surfactant replacement therapy or had long intubation for more hours. | NR | Post-extubation |
| 2014, Ciuffini F et al^26^ | Itália | CPAP: 4-6 cmH _2_O;  HFNC: 4-6 L/min. | Y | GA 29 and 36 weeks;  mild to moderate RD; Informed parental consent;  FiO2 > 0.35-0.40 to maintain  an SpO2 between 85-93% and / or dyspnea intubated and treated with INSURE | Congenital mal formations and IVH severe, not possible to obtain consent from the parents. | NR | Primary support |
| 2013, Yoder B A et al^27^ | EUA and China | HFNC: infant weight.  CPAP: 5-6 cm H_2_O. | Y | >1000 g and GA 28 weeks. | Air leak syndrome; abnormalities of upper and lower airways; serious abdominal, cardiac, or respiratory malformations. | Oxygen reduction test | Post-extubation |
| 2013, Collins C L et al^28^ | Australia | HNFC: 4 L/min  CPAP: 8 cm/ H _2_O | Y | GA< 32 weeks, endotracheal intubation and positive pressure ventilation | Upper airway obstruction, congenital airway malformations, or major cardiopulmonary malformations. | 36 weeks PMA | Post-extubation |
| 2013, Manley B J et al^29^ | Australia | HFNC: 5-6 L/min  CPAP: 7 cm/ H_2_O | Y | <32 weeks, MV through an endotracheal tube, and were scheduled to undergo extubation for the first time to noninvasive respiratory support. | GA>36 weeks at the time of extubation,  major congenital anomaly. | 36 weeks PMA | Post-extubation |

**BPD:** Bronchopulmonary dysplasia **RCT:** Randomized Clinical Trials **HFNC:** High flow nasal cannula **CPAP:** Continuous Positive Airway Pressure **NICHD:** National Institute of Child Health and Human Development **PMA:** Postmenstrual age **NIPPV:** Intermittent positive pressure ventilation **PT:** preterm **GA:** gestational age **SDR:** syndrome distress respiratory **DR:** delivery room **MV:** mechanical ventilation **IVH:** intraventricular hemorrhage
