# Supplementary material for: Can high-flow nasal cannula reduce the risk of bronchopulmonary dysplasia compared with CPAP in preterm infants? A systematic review and meta-analysis
Source: BMC Pediatr. 2021 Sep 16;21:407. doi: 10.1186/s12887-021-02881-z (PMC8444598; doi:10.1186/s12887-021-02881-z)
Supplement: Supplementary file 4 — Additional file 4. Characteristics of the participants enrolled in studies. [file 12887_2021_2881_MOESM4_ESM.docx]

**Additional file 4- Characteristics of the participants enrolled in studies.**

| Year, Author | Gestational age HFNC  (mean ± SD, weeks) | Gestational age CPAP  (mean ± SD, weeks) | Gender male/female n (%) CPAP | Gender male/female n (%) HFNC | Birth weight (mean, ±SD, g) nCPAP | Birth weight (mean, ±SD, g) HFNC | Surfactant  (Y/N) | Associated diseases |
| --- | --- | --- | --- | --- | --- | --- | --- | --- |
| 2020, Chen J et al^11^ | 27.2 ± 2.8 | 27.5 ± 3.2 | M: 29 (63.4) | M: 30 (62.5) | 794 ± 31.0 | 827± 23.0 | NR | HFNC:  4- 7 (14.58); 6- 17 (35.42); 2- 16 (33.33); 5- 5 (10.42)  CPAP: 4- 7 (15.21); 6- 18 (39.13); 2- 16 (34.78); 5- 13 (28.26) |
| 2020, Leibel S et al^17^ | 25.4 (24.9, 26.7)  Median (IQR) | 25.15 (24.4, 26.5)  Median (IQR) | F: 50 (6/12) | F: 61.54 (8/13) | 780 (630, 945)  Median (IQR) | 790 (730, 840)  Median (IQR) | Y | CPAP % (n/N): 2- 50 (6/12); 5- 8.33 (1/12); 6- 41.67 (5/12); 4- 0 (0/12)  HFNC: 2- 53.8 (7/13); 5- 0 (0/12); 6- 38.46 (5/13); 4- 7.69 (1/13) |
| 2019, Demirel G et al^18^ | 31.2 (2.3)  Median (IQR) | 31.0 (1.8)  Median (IQR) | F: 25 (46.2) | F: 23 (43.3) | 1505 (580)  Median (IQR) | 1570 (455)  Median (IQR) | Y | HFNC: 2- 5 (9.4%); 1- 6 (11.3%); 4- 2 (3.7%); 5- 1 (1.8%); 6-2 (3.7%); 3-2 (3.7%)  CPAP: 2- 6 (11.1%); 1-7 (12.9%); 4-1 (1.8%); 5-1 (1.8%); 6-1 (1.8%); 3-1 (1.8%) |
| 2019, Shokouhi M et al^19^ | 31.80 ± 1.19 | 32.63 ± 1.61 | M: 22 (73.3) | 14 (46.7) | 1840.73± 637.92 | 1743.83 ± 317.35 | N | HFNC: 3- 0 (0); 4-1 (3.3); 6- 0 (0); 2- 6 (20)  CPAP: 3- 0 (0); 4- 0 (0); 6- 2 (6.7); 2- 3 (10) |
| 2018, Murki S et al^20^ | 31.8 ± 1.9 | 31.6 ± 2.2 | M: 77 (55) | M: 73 (55) | 1,642 ± 437 | 1,632 ± 431 | Y | CPAP: 5- 0; 4- 1 (0.7) ;6- 7 (5.0)  HFNC: 5- 2 (1.5); 4- 0; 6- 6 (4.5) |
| 2018, Farhat A A et al^21^ | 31.3 (1.9)  Median (IQR) | NIPPV =  31.8 (1.7)  31.1(2)  Median (IQR) | M= 64.2%  NIPPV-M=  45.3% | M= 61.1% | 1650(486)  NIPPV=1622 (437)  Median (IQR) | 1624(425)  Median (IQR) | Y | NR |
| 2017, Soonsawad S et al^22^ | 27.5 (26, 30)  Median (IQR) | 28 (25, 29.5)  Median (IQR) | M: 17 (68.0) | M: 11 (45.8) | 980 (740, 1237)  Median (IQR) | 990 (800, 1333)  Median (IQR) | Y | HFNC: 5- 8.3%; 6- 16%; 4- 8%  CPAP: 5- 88%; 6- 30%; 4- 4% |
| 2017, Shin J et al^10^ | 32.5 ± 1.5 | 33.0 ± 1.2 | M: 24 (55.8) | M: 23 (54.8) | 1,996±374 | 2,058±371 | N | HFNC: 4- 0 (0.0); 5- 0 (0.0)  CPAP: 4-0 (0.0); 5- 0 (0.0) |
| 2016, Lavizzari A et al^23^ | 33.1 ± 1.9 | 33.0 ± 2.1 | M: 83 | M: 75 | 1908 ± 528 | 1968 ±581 | Y | NR |
| 2016, Kadivar M et al^24^ | 31.52 | 31.33 | M: 14/27 | M: 14/27 | 1,601 | 1,642 | Y | NR |
| 2014, Ciuffini F et al^26^ | 33 ± 2 | 33 ± 2 | 51/41 | 44/41 | 1936 (± 537) | 1922 (± 589) | Y | NR |
| 2013, Yoder B A et al^27^ | 33.5 ± 3.6 | 33.2 ± 3.2 | M: 137 | M: 137 | 2108 ±782 | 2201 ±816 | Y | HFNC: 5- 2(1)  CPAP: 5- 4(2) |
| 2013, Collins C L et al^28^ | 27.9 (1.95)  Median (IQR) | 27.6 (1.97)  Median (IQR) | M: 41 | M: 33 | 1105 (374)  Median (IQR) | 1123 (317)  Median (IQR) | Y | NR |
| 2013, Manley B J et al^29^ | 27.7 ± 2.1 | 27.5 ± 1.9 | M: 72 (47.7) | M: 89 (58.6) | 1044 ± 327 | 1041±338 | Y | HFNC: 2- 64 (42.1); 5-3 (2.0); 4- 3 (2.0)  CPAP: 2- 64 (42.4); 5- 7 (4.6); 4- 8 (5.3) |

The data are presented as media ± SD or n (%) * Median (inter quartile range) **Associated diseases: 1-**Sepsis Precoce; **2-** Patent ductus Arteriosus; **3-**pneumothorax (PNX); **4-** intraventricular hemorrhage (IVH), **5-** infections necrotizing enterocolitis (NEC); **6-**retinopathy of prematurity (ROP)
