# Supplementary figures and images for: Can high-flow nasal cannula reduce the risk of bronchopulmonary dysplasia compared with CPAP in preterm infants? A systematic review and meta-analysis
Source: BMC Pediatr. 2021 Sep 16;21:407. doi: 10.1186/s12887-021-02881-z (PMC8444598; doi:10.1186/s12887-021-02881-z)

Additional file 5- Assessment of risk of bias of included clinical trial studies.


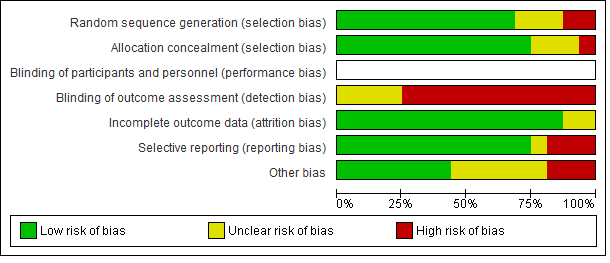

Supplement: Supplementary file 5 — Additional file 5. Assessment of risk of bias of included clinical trial studies. [file 12887_2021_2881_MOESM5_ESM.docx]
