# Supplementary material for: Can high-flow nasal cannula reduce the risk of bronchopulmonary dysplasia compared with CPAP in preterm infants? A systematic review and meta-analysis
Source: BMC Pediatr. 2021 Sep 16;21:407. doi: 10.1186/s12887-021-02881-z (PMC8444598; doi:10.1186/s12887-021-02881-z)
Supplement: Supplementary file 6 — Additional file 6. GRADE scale. [file 12887_2021_2881_MOESM6_ESM.docx]

Additional file 6- GRADE scale.

|  | | | | | | | **№ of patients** | | **Effect** | | **Certainty** | **Importance** |
| --- | --- | --- | --- | --- | --- | --- | --- | --- | --- | --- | --- | --- |
| **№ of studies** | **Study design** | **Risk of bias** | **Inconsistency** | **Indirectness** | **Imprecision** | **Other considerations** | **[HFNC]** | **[CPAP]** | **Relative (95% CI)** | **Absolute (95% CI)** |  |  |

**Bronchopulmonary dysplasia (follow up: range 28 days to 4 months; assessed with: days of oxygen utilization)**

| 13 | randomised trials | serious ^a^ | not serious | very serious  b | very serious ^c^ | none | 133/1014 (11.1%) | 145/1024 (14.1%) | **RR 1.10**  (0.90 to 1.34) | **12 more per 1.000**  (from 13 fewer to 42 more) | ⨁◯◯◯  VERY LOW | CRITICAL |
| --- | --- | --- | --- | --- | --- | --- | --- | --- | --- | --- | --- | --- |

**Nasal injury (assessed with: evaluation with protocol)**

| 7 | randomised trials | not serious | serious ^d^ | very serious  e | serious ^f^ | publication bias strongly suspected  g | 231/672 (34.4%) | 305/683 (44.7%) | **RR 2.37**  (0.92 to 6.13) | **612**  **more per 1.000**  (from 36 fewer to 1.000  more) | ⨁◯◯◯  VERY LOW | IMPORTANT |
| --- | --- | --- | --- | --- | --- | --- | --- | --- | --- | --- | --- | --- |

**Air leak syndrome (pneumothorax) (follow up: range 1 days to; assessed with: RX tx)**

| 10 | randomised trials | not serious | serious ^h^ | very serious  i | very serious ^j^ | none | 15/801 (1.8%) | 17/815 (2.0%) | **RR 1.06**  (0.52 to 2.14) | **1 more per 1.000**  (from 9 fewer to 19 more) | ⨁◯◯◯  VERY LOW | IMPORTANT |
| --- | --- | --- | --- | --- | --- | --- | --- | --- | --- | --- | --- | --- |

**CI:** Confidence interval; **RR:** Risk ratioExplanations

1. the studies do not describe the evaluator's blinding of the outcomes; three studies out of 14 did not provide a description of the randomization sequence and allocation method.
2. of the 14 studies, only 8 describe how the outcome was defined; of the 14 studies, only 5 did not limit the GI of premature infants.
3. small effect measure, however broad 95% CI (0.90-1.32)
4. 95% broad CI (0.92-6.13)
5. of the 07 studies analyzed, only 01 described the protocol used to assess the outcome.
6. wide IC of the measure of effect.
7. asymmetry between studies assessed by the funnel plot.
8. 95% broad (0.57-1.90) and asymmetric CI.
9. of the 11 studies evaluated, only 03 presented the outcome assessment method.
10. 95% broad CI (> 1.25) and small number of events (0.57-1.90).
